# Case Report: De Winter electrocardiographic pattern associated with acute total left main coronary artery occlusion

**DOI:** 10.3389/fcvm.2026.1865719

**Published:** 2026-08-18

**Authors:** Jian Wang, Peiran Yan, Faming Ding, Yiwen Wang, Min Feng

**Affiliations:** Department of Cardiology, Shandong Medical and Pharmaceutical University Hospital, Binzhou, China

**Keywords:** acute coronary syndrome, case report, de Winter ECG pattern, left main coronary artery, percutaneous coronary intervention

## Abstract

**Background:**

The de Winter electrocardiograph (ECG) pattern is a well-established ST-elevation myocardial infarction (STEMI) equivalent, most commonly associated with acute occlusion of the proximal left anterior descending (LAD) artery.

**Case presentation:**

A 54-year-old man presented to the emergency department with two hours of chest pain, sweating, and nausea. The 12-lead ECG showed upsloping J-point depression in leads V2–V6 and lead I, with tall, symmetrical T waves in leads V2–V5, ST-segment elevation in lead aVR, and ST-segment depression in the inferior leads, consistent with the de Winter pattern. Emergency coronary angiography revealed total occlusion of the left main coronary artery (LMCA), and a drug-eluting stent was implanted. Due to refractory heart failure and worsening hemodynamics, the patient underwent intra-aortic balloon pump (IABP) and extracorporeal membrane oxygenation (ECMO) treatment. The patient was discharged 40 days later.

**Conclusion:**

This case demonstrates that the de Winter ECG pattern may occasionally indicate LMCA occlusion rather than isolated LAD occlusion. In the setting of a de Winter ECG pattern accompanied by inferior lead ST-segment depression and ST-segment elevation in lead aVR, clinicians should raise clinical suspicion for LMCA occlusion—particularly in hemodynamically unstable patients—to facilitate timely and comprehensive intervention.

## Introduction

The de Winter ECG pattern (1) is a well-established STEMI equivalent with a high predictive value (95.2%–100%) (2) for acute proximal LAD occlusion, necessitating urgent revascularization. However, emerging evidence has linked this pattern to occlusions of other coronary territories—including the right coronary artery (RCA) (3), left circumflex artery (LCX) (4), and even diagonal branches (5)—as well as to non-atherosclerotic conditions such as myocarditis (6) and type A aortic dissection (7).

Left main coronary artery (LMCA) occlusion is a rare, often fatal event, yet its association with the de Winter pattern remains poorly documented and easily overlooked. Traditionally, LMCA occlusion presents with distinct ECG findings rather than the de Winter morphology. Here, we report a case of total LMCA occlusion presenting with a de Winter ECG pattern, discuss the diagnostic challenges, and emphasize the need to consider this lethal etiology, particularly in unstable patients.

## Case presentation

**Figure 1:**
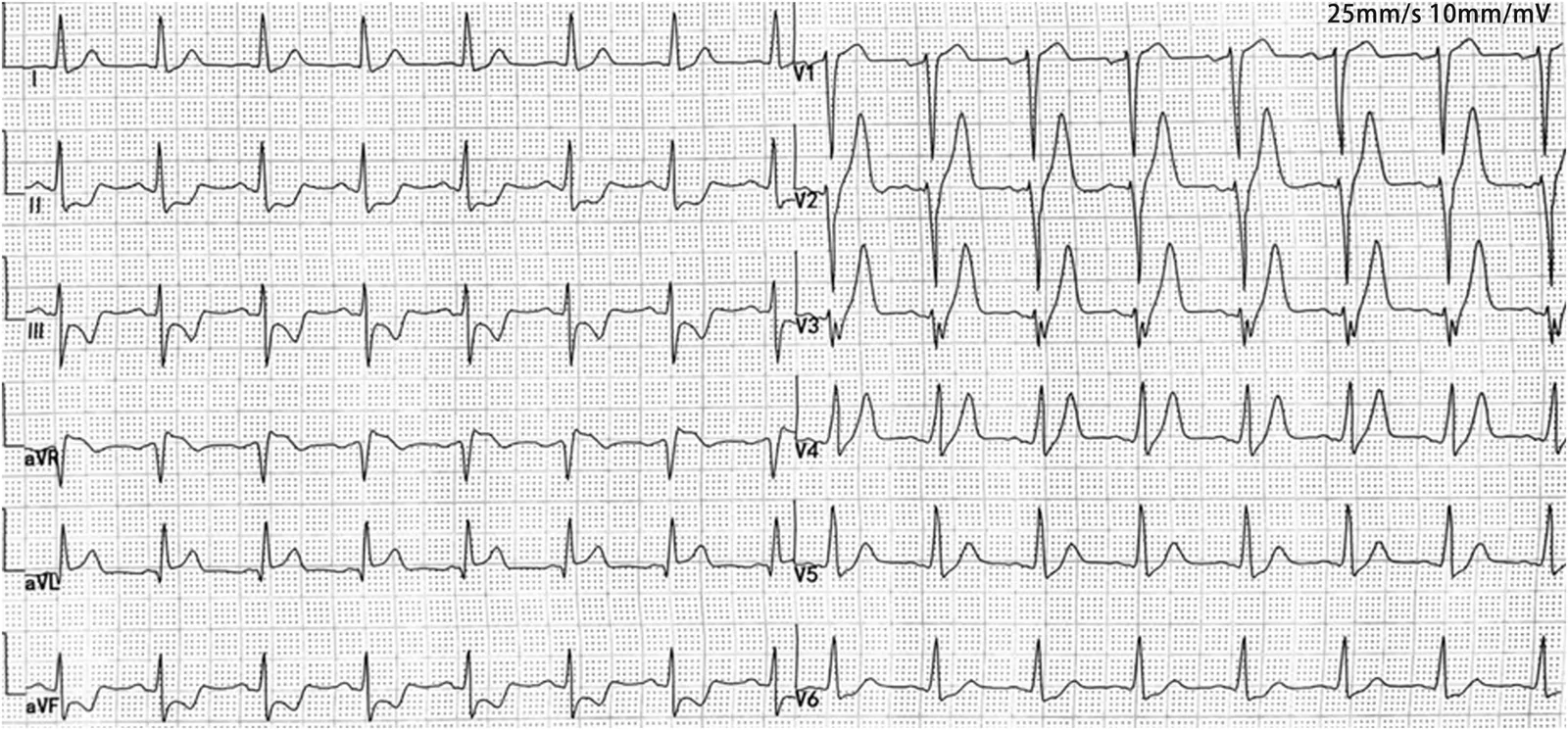
The 12-lead ECG shows sinus rhythm at 90 bpm, QRS duration 104 ms, with precise distribution and amplitude of ST-segment and T-wave abnormalities: ST-segment depression of 0.1–0.4 mV in leads I and V_2_–V_6_, followed by upright symmetrical T waves of 0.3–1.1 mV; ST-segment elevation in leads aVR and V_1_ (aVR > V_1_); downsloping ST-segment depression of 0.1–0.3 mV in the inferior leads; and upsloping ST-segment elevation of 0.1 mV in lead aVL.

**Figure 2:**
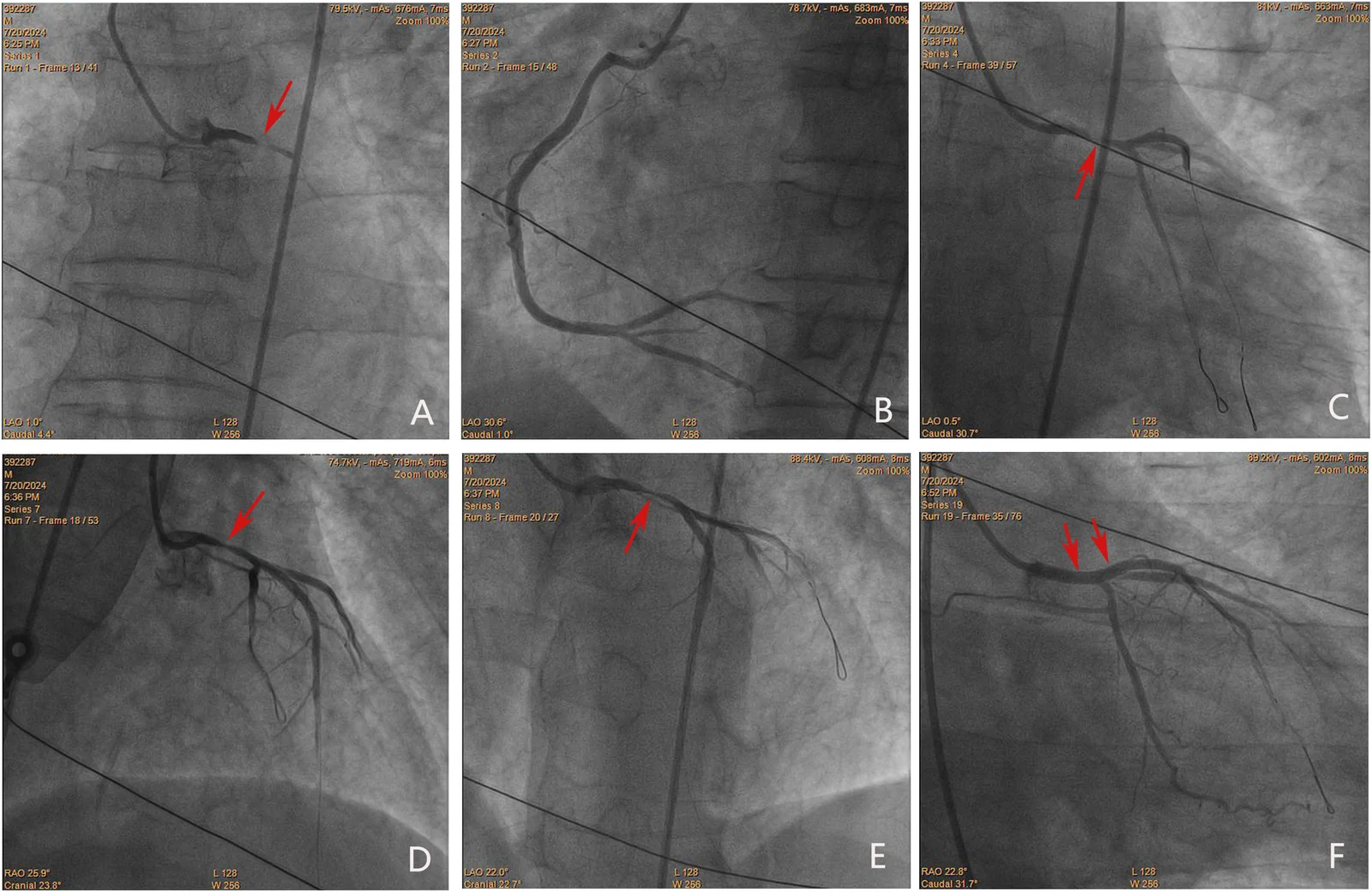
**(A)** Coronary angiogram shows mid-segment occlusion of the LMCA with antegrade TIMI grade 0 flow, and the red arrow indicates the site of occlusion. **(B)** The proximal right coronary artery (RCA) showed no significant obstructive coronary disease. **(C)** After guidewire passage through the occluded segment, antegrade TIMI grade 1 flow was observed in the LAD and antegrade TIMI grade 2 flow in the LCX, and the red arrow indicates the lesion site. **(D,E)** Following balloon dilatation, residual stenosis of the left main coronary artery was 70% (red arrow), flow in the LAD improved to TIMI grade 2, and flow in the LCX improved to antegrade TIMI grade 3. **(F)** Drug-eluting stent was subsequently implanted at the LM–LAD lesion (red arrow), antegrade TIMI grade 3 flow was observed in the LAD and LCX.

A 54-year-old man with no significant medical history presented to the emergency department with persistent chest pain accompanied by sweating and nausea for two hours. The patient had a history of smoking, hypertension and dyslipidemia. There was no history of diabetes, family history or other cardiovascular risk factors. The 12-lead ECG revealed an upsloping ST-segment depression of up to 4 mm at the J point in leads V_2_–V_6_ and lead I; tall, symmetrical T waves in leads V_2_–V_5_; 2 mm J point elevation in lead aVR; and a 2 mm ST-segment depression in the inferior leads, suggestive of the de Winter ECG pattern (Figure 1). On arrival, the patient's heart rate was 90 beats/min, and blood pressure was 80/50 mmHg. Crackles were auscultated in the lungs, and heart sounds were regular without murmurs, rubs, or gallops. The patient received loading-dose antiplatelet therapy with aspirin enteric-coated tablets 300 mg and ticagrelor 180 mg, together with rosuvastatin 10 mg. Emergency coronary angiography revealed total occlusion of the LMCA with antegrade TIMI grade 0 flow (Figure 2A), no collateral channels, and no significant obstructive coronary disease in the RCA (Figure 2B). After guidewire passage through the occluded segment, antegrade TIMI grade 1 flow was observed in the LAD and antegrade TIMI grade 2 flow in the LCX (Figure 2C). Following balloon dilatation, flow in the LAD improved to TIMI grade 2, and flow in the LCX improved to antegrade TIMI grade 3 (Figures 2D,E). A 3.5 mm × 21 mm drug-eluting stent was implanted in the LMCA (Figure 2F). Ventricular fibrillation developed during the procedure and was successfully terminated by defibrillation; an intra-aortic balloon pump (IABP) was inserted due to hypotension. The time from admission to balloon dilation was 56 min. Transthoracic echocardiography demonstrated a left ventricular ejection fraction (LVEF) of 39% and hypokinesia of the anterior wall. Laboratory tests showed elevated troponin I levels, with a peak value of 100 ng/mL (normal value <0.03 ng/mL) at 24 h after presentation. On admission, the N-terminal pro-brain natriuretic peptide (NT-pro BNP) level was recorded at 7,640 pg/mL, which had increased to 13,400 pg/mL by the following day. Due to refractory heart failure and worsening hemodynamics, the patient underwent extracorporeal membrane oxygenation (ECMO) treatment on July 24, 2024. After 7 days of application, the patient was weaned off the machine (VA-ECMO) on July 31, 2024. During hospitalization, the patient also suffered from serious infections and other illnesses. The patient was discharged 40 days later. The patient expressed satisfaction with the therapeutic outcomes. Over the follow-up period, he experienced one hospitalization for heart failure, with no additional admissions thereafter. His New York Heart Association (NYHA) functional class was II. Transthoracic echocardiography revealed left ventricular aneurysm formation, segmental wall motion abnormalities, and a LVEF ranging between 35% and 44%.

## Discussion

We report a case of total left main coronary artery (LMCA) occlusion presenting with the de Winter ECG pattern—an association not originally described by de Winter et al. and rarely highlighted since. Although this pattern is a highly specific marker of proximal left anterior descending (LAD) occlusion, our case adds to the limited evidence that it may also signal more catastrophic lesions, including LMCA occlusion.

Acute total LMCA occlusion is a rare, life-threatening event often complicated by cardiogenic shock and malignant arrhythmias. Two classic ECG patterns have been described (8): a STEMI pattern (STE in V_2_–V_6_, I, aVL, sometimes with right bundle-branch block and left anterior fascicle block) and a non-STEMI pattern (widespread ST depression in ≥6 leads with STE in aVR and V1, aVR > V1). Notably, the de Winter pattern is not among these classic presentations, consistent with de Winter et al.'s original observation (1) that their pattern showed no evidence of left main involvement.

Several subsequent reports have attempted to link the de Winter pattern to LMCA occlusion. Zhan (9) described a case attributed to LMCA occlusion, but the ECG showed widespread ST depression—downsloping ST depression in leads III and aVF, and horizontal ST depression in leads I, II, and V_2_ through V_5_—with aVR/V_1_ elevation, features typical of the classic LMCA pattern rather than true de Winter morphology. ECG signs of global ischemia (ST depression in 9 leads and ST elevation in aVR) suggest the possibility of left main coronary artery disease. Liu (10) reported three cases of the de Winter ECG pattern associated with LMCA occlusion. In all three patients, the admission ECGs demonstrated ST-segment depression of 0.1–0.8 mV in leads V_2_–V_6_, maximal in V_4_–V_5_, with tall, positive symmetrical T waves. Two patients showed upsloping ST-segment depression of 0.1–0.3 mV in the inferior leads; and two patients presented with concomitant ST-segment elevation in leads aVR and V_1_ (with aVR > V_1_). one patient showed ST elevation only in lead aVR, without V_1_ involvement. Accordingly, they believe that the de Winter ECG pattern with a widespread upsloping ST depression in V_2_–V_6_ centered around V_5_ may indicate total LM occlusion. Kashou et al. (11) reported a lateral variant of the de Winter pattern in a patient with distal left main stenosis and concomitant ostial LAD occlusion. The ECG showed widespread upsloping ST depression with tall symmetrical T waves in leads V_2_–V_6_, I, and II, with maximal deviation shifting to lead V_5_—suggesting concurrent lateral wall ischemia. This case further supports that broader territorial involvement should raise suspicion for left main or combined LAD/LCX disease rather than isolated mid-LAD occlusion.

Our case differed somewhat from the above reports. The ECG in our patient revealed ST-segment depression of 0.1–0.4 mV in leads I and V_2_–V_6_, with the maximal depression occurring in lead V_3_ rather than V_4_/V_5_, followed by upright, symmetrical T waves—thus fully satisfying the standard de Winter criteria as summarized by Morris and Brod (2). Concurrently, there was ST-segment elevation in leads aVR and V_1_ (aVR > V_1_), along with downsloping ST-segment depression of 0.1–0.3 mV in the inferior leads and upsloping ST-segment elevation of 0.1 mV in lead aVL. In addition, the three patients reported by Liu were hemodynamically stable, whereas this patient presented with cardiogenic shock. Of note, in our case, the de Winter ECG pattern coexisted with extensive ST-segment depression in the inferior and lateral leads, as well as ST-segment elevation in lead aVR, which may suggest more severe global subendocardial ischemia, potentially secondary to LMCA involvement rather than isolated LAD occlusion. Whether this patient's presentation represents a pure de Winter syndrome pattern or a fusion of the de Winter pattern and a left main disease pattern remains debatable.

LMCA occlusion abruptly halts flow to both the LAD and LCX, causing global left ventricular subendocardial ischemia. The vulnerable subendocardium, with high metabolic demand and low perfusion pressure, develops severe injury while the epicardium remains relatively spared, creating a transmural injury gradient. This generates a net injury current directed from the ischemic endocardium toward the epicardium, producing widespread ST depression in leads I, V_2_–V_6_, and the inferior leads. Lead aVR, facing the right superior shoulder, reciprocally mirrors the inferolateral LV injury, resulting in ST elevation (aVR > V_1_) (12), confirming a predominantly superior and rightward injury vector—a hallmark of global ischemia. The upsloping ST elevation in aVL reciprocally corresponds to downsloping ST depression in the inferior leads. Meanwhile, global ischemia prolongs repolarization and increases dispersion, producing tall, symmetrical upright T waves in leads with ST depression, reflecting ischemic repolarization abnormalities distinct from strain patterns. Collectively, this ECG constellation represents the electrophysiological signature of severe global LV subendocardial ischemia, driven by a massive injury vector directed away from the diffusely injured subendocardium, with aVR specifically mirroring the extent of ischemic burden (13).

Thus, to date, no reliable ECG feature definitively distinguishes LMCA from LAD occlusion within the de Winter pattern. The characteristics of this pattern when associated with LMCA occlusion remain unclear, and further case accumulation with quantitative analysis is needed.

## Conclusion

This case demonstrates that total LMCA occlusion can also present with the de Winter ECG pattern. At present, no reliable ECG feature within this pattern definitively differentiates LMCA from LAD occlusion. The presence of a de Winter ECG pattern with concomitant ST-segment depression in the inferior leads and ST-segment elevation in lead aVR, or with aVR elevation greater than that in lead V_1_, should raise clinical suspicion for LMCA occlusion—particularly in hemodynamically unstable patients—and should consider mechanical circulatory support (e.g., IABP, ECMO). Further case accumulation and quantitative analysis are warranted to better characterize the de Winter pattern associated with LMCA occlusion.

## Data Availability

The original contributions presented in the study are included in the article/Supplementary Material, further inquiries can be directed to the corresponding author.
